# Effect of tumor-associated macrophages on lncRNA PURPL/miR-363/PDZD2 axis in osteosarcoma cells

**DOI:** 10.1038/s41420-021-00700-z

**Published:** 2021-10-22

**Authors:** Fan He, Guoming Ding, Wu Jiang, Xiaoliang Fan, Liulong Zhu

**Affiliations:** Department of Orthopedics, Affiliated Hangzhou First People’s Hospital, Zhejiang University School of Medicine, 261 HuanSha Road, ShangCheng, HangZhou, ZheJiang 310006 China

**Keywords:** Cell biology, Cancer

## Abstract

Tumor-associated macrophages (TAMs) are known to participate in osteosarcoma (OS) progression. As demonstrated in our previous research, miR-363 played a tumor inhibitory effect in OS cells via lowering the PDZ domain containing 2 (PDZD2) expression. The regulatory roles of TAMs on miR-363/PDZD2 and the internal mechanism relating to long noncoding RNA p53 upregulated regulator of P53 levels (lncRNA PURPL) are examined in this study. TAM-like macrophages were formed by inducing CD14^+^ peripheral blood mononuclear cells (PBMCs). The TAMs migration was detected after MG-63 cells transfected with miR-363 mimics or inhibitors. We then analyzed the regulatory activity of PURPL on miR-363 expression. We also tested the influences of PURPL overexpression/knockdown on MG-63 cell proliferation, migration, invasion, and epithelial-mesenchymal transition (EMT), as well as TAMs migration. Silence in PDZD2 expression was used to confirm the effects of PURPL on MG-63 cells. We successfully induced TAM-like macrophages. MG-63 cells transfecting miR-363 mimics suppressed TAMs migration while transfecting a converse effect was seen in miR-363 inhibitor. TAMs raised PURPL expression in MG-63 cells, which was an upstream regulator of miR-363. Along with TAMs migration, PURPL overexpression promoted MG-63 cell proliferation, migration, invasion, and EMT. An opposite influence was seen due to the PURPL knockdown. The silence of PDZD2 weakened the influences of PURPL overexpression on MG-63 cells and TAMs migration. On modulating the PURPL/miR-363/PDZD2 axis, TAMs-promoted OS development might be achieved.

## Introduction

Osteosarcoma (OS) is a primary malignant bone sarcoma that constantly occurs in the long bones of children and adolescents [1]. The long-term survival rate in OS patients remains 10–20% due to the complexity of the pathogenesis and the high rate of tumor metastasis [2]. Macrophages that originate from the precursor cells in the bone marrow are a key component of the human innate immune system [3]. Several research works in recent years have demonstrated some macrophages, named tumor-associated macrophages (TAMs) that existed in the tumor microenvironment and contributed to the proliferation and metastasis of tumor [4, 5]. Cersosimo et al. [6] reported that tumor metastasis and unsatisfied prognosis of OS patients were related to the increased infiltration of M2-like TAMs infiltration. Han et al. [7] indicated that via modulating the cyclooxygenase-2 (COX-2)/signal transducer and activator of transcription 3 (STAT3) signaling, TAMs facilitated the epithelial-mesenchymal transition (EMT) and lung metastasis of OS. More investigations are demanded to further find the internal regulatory mechanism of TAMs on OS growth and metastasis.

The gene-coding activity is not present in long noncoding RNAs (lncRNAs) [8]. As reported in earlier literature, lncRNAs took part in transcriptional and post-transcriptional modulation via cross-talking with other RNAs [9]. They are verified to closely relate to cancer occurrence and development [10]. As indicated by Su et al. [11], macrophage-derived C−C motif chemokine ligand 18 (CCL18) promoted OS proliferation and migration via enhancing lncRNA urothelial carcinoma-associated 1 (UCA1) expression. As demonstrated by Yang et al. [12], lncRNA RP11-361F15.2 promoted OS progression via microRNA-30c-5p/cytoplasmic polyadenylation element-binding protein 4 (CPEB4)-mediated suppression of M2-like polarization of TAMs. LncRNA p53 upregulated regulator of P53 level (PURPL) is responsible for regulating p53 levels in cells via associating with MYB binding protein 1A (MYBBP1A), which is reported to inhibit basal p53 levels and accelerate tumorigenicity in colorectal cancer [13]. Zhang et al. [14] and Moridi et al. [15] demonstrated that dis-regulation of PURPL could serve as a potential tumor biomarker in epithelial ovarian cancer and gastric cancer.

MicroRNAs (miRs) are also regulatory RNAs in cells that exert key regulatory functions in the tumorigenesis of multiple cancers, including OS [16]. As indicated in our previous experiments, miR-363 exerted tumor inhibitory activity in OS cells via suppressing the PDZ domain containing 2 (PDZD2) expression [17]. miRs, including miR-363, were generally modulated by lncRNAs [18, 19]. Our aim in this research was to investigate whether lncRNAs (especially PURPL) were regulated by TAMs and took part in the proliferation and metastasis of OS cells by modulating the miR-363/PDZD2 axis. To further comprehend the internal mechanism regarding the promoting function of TAMs on OS development, our research findings will be of use.

## Results

### TAM-like macrophages successfully induced

**Fig. 1:**
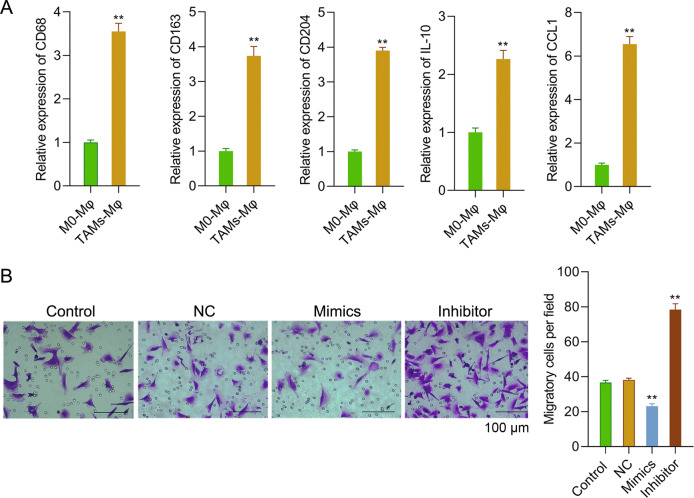
TAM-like macrophages were successfully induced, and different expressions of miR-363 in MG-63 cells influenced TAMs migration. **A** CD14^+^ PBMCs were stimulated by hM-CSF and an osteosarcoma-conditioned medium. qPCR was used to test CD68, CD163, CD204, IL-10, and CCL1 expressions in cells. **B** TAMs were added into the upper-chamber, while MG-63 cells transfecting miR-363 mimics or inhibitors were put into the lower-chamber. Forty-eight hours later, the migration of TAMs was detected. **P < 0.01 vs. M0-Mφ or NC group.

hM-CSF and 50% osteosarcoma-conditioned medium were used to treat CD14^+^ PBMCs to form TAM-like macrophages. Figure 1A showed that after hM-CSF and 50% osteosarcoma-conditioned medium treatment (*P* < 0.01), the expression levels of macrophage-associated molecules including CD68, CD163, CD204, IL-10, and CCL1 in CD14^+^ PBMCs were significantly increased, which showed that TAMs-like macrophages were successfully induced.

### MG-63 cells with different miR-363 expressions influenced TAMs migration

MG-63 cells were subjected to miR-363 mimics or inhibitor transfection to explore the influence of miR-363 abnormal expression on TAMs migration. As shown in Fig. 1B, miR-363 mimics transfection notably suppressed TAMs migration (*P* < 0.01), while miR-363 inhibitor promoted TAMs migration (*P* < 0.01). As shown in these results, MG-63 cells with different miR-363 expression influenced TAMs migration.

### TAMs upregulated the expression of several lncRNAs in MG-63 cells

**Fig. 2:**
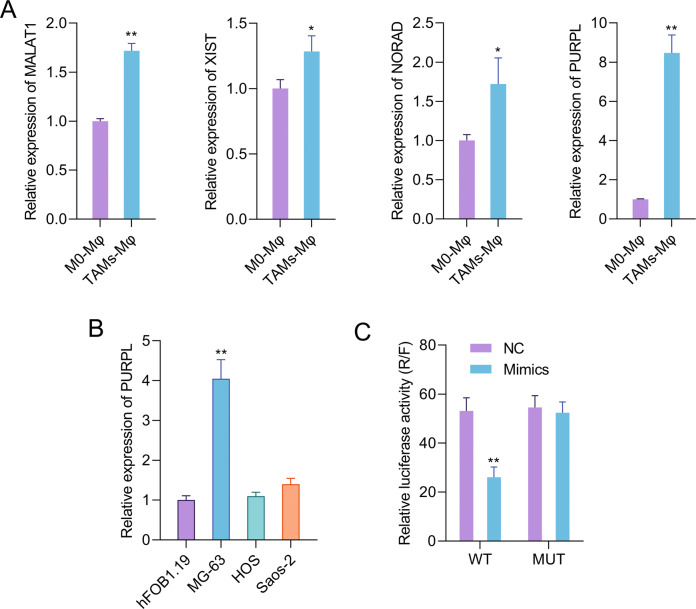
TAMs upregulated the expression of several lncRNAs in MG-63 cells. **A** TAMs were cultivated into the upper- chamber, while MG-63 cells were put into the lower- chamber. Forty-eight hours later, the expression of MALAT1, XIST, NORAD, and PURPL in MG-63 cells were tested via qPCR. **B** qPCR was used to test PURPL expression in hFOB1.19, MG-63, HOS, and Saos-2 cells. **C** MG-63 cells were subjected to miR-363 mimics and PURPL-WT (or PURPL-MUT) transfection. The relative luciferase activity (R/F) was detected. *P < 0.05, **P < 0.01 *vs*. hFOB1.19, M0-Mφ, or NC group.

As discovered in bioinformatics analysis, a lot of lncRNAs could bind to miR-363. Among these, high binding coefficients were seen in MALAT1, XIST, NORAD, and PURPL. To detect MALAT1, XIST, NORAD, and PURPL expression in MG-63 cells, qPCR was applied after TAMs co-culture. Figure 2A showed that the expressions of MALAT1, XIST, NORAD, and PURPL in MG-63 cells all remarkably increased after TAMs co-culture (*P* < 0.05 or *P* < 0.01). PURPL expression had the most obvious change, which was selected for subsequent experiments. Figure 2B showed that compared to normal human osteoblastic hFOB1.19 cells, PURPL expression was higher in osteosarcoma MG-63 cells (*P* < 0.01). As shown in Fig. 2C, PURPL-WT and miR-363 mimic co-transfection significantly lowered the relative luciferase activity (R/F, *P* < 0.01), which implied that PURPL was a potential upstream regulator of miR-363 and attended to the modulation of MG-63 cell function.

### PURPL attended to modulation of MG-63 cell proliferation, migration, and invasion

**Fig. 3:**
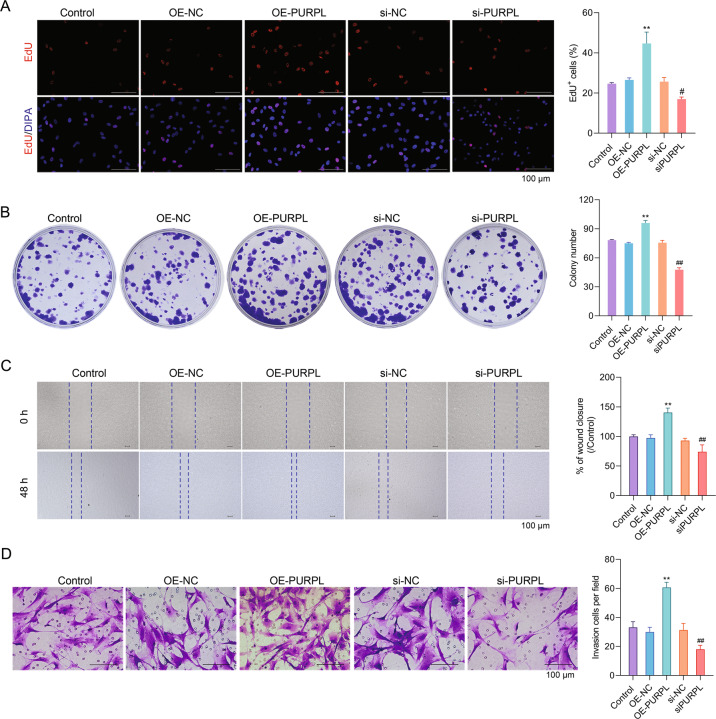
PURPL attended to the modulation of MG-63 cell proliferation, migration, and invasion. OE-PURPL or si-PURPL was transfected into MG-63 cells. Proliferation (**A**, **B**), migration (**C**), and invasion (**D**) of MG-63 cells were detected via EdU incorporation assay, colony formation assay, cell scratch assay, and transwell assay, respectively. **P < 0.01 vs. OE-NC group. ^*#*^*P* < 0.05, ^*##*^*P* < 0.01 vs. si-NC group.

MG-63 cells were subjected to OE-PURPL or si-PURPL transfection to investigate the possible regulatory effects of PURPL overexpression, or knockdown on MG-63 cell proliferation, migration, invasion, and EMT. Figure 3A displayed that the EdU^+^ cells (%) in the OE-PURPL group were notably high than those in the OE-NC group (*P* < 0.01). Compared to the si-NC group, the EdU^+^ cells (%) in the si-PURPL group were remarkably low (*P* < 0.05). The colony number of MG-63 cells was also raised by OE-PURPL transfection (Fig. 3B, *P* < 0.01), while si-PURPL transfection had an opposite influence (*P* < 0.01). Moreover, Fig. 3C, D presented that OE-PURPL transfection notably promoted MG-63 cell migration and invasion, as evidenced by the increased percentage of wound closure invasion cells per field (*P* < 0.01). Contrary influence was exerted by si-PURPL transfection (*P* < 0.01). As proposed by these results, PURPL modulated MG-63 cell proliferation, migration, and invasion.

### PURPL modulated MG-63 cell EMT, as well as miR-363 and PDZD2 expression

**Fig. 4:**
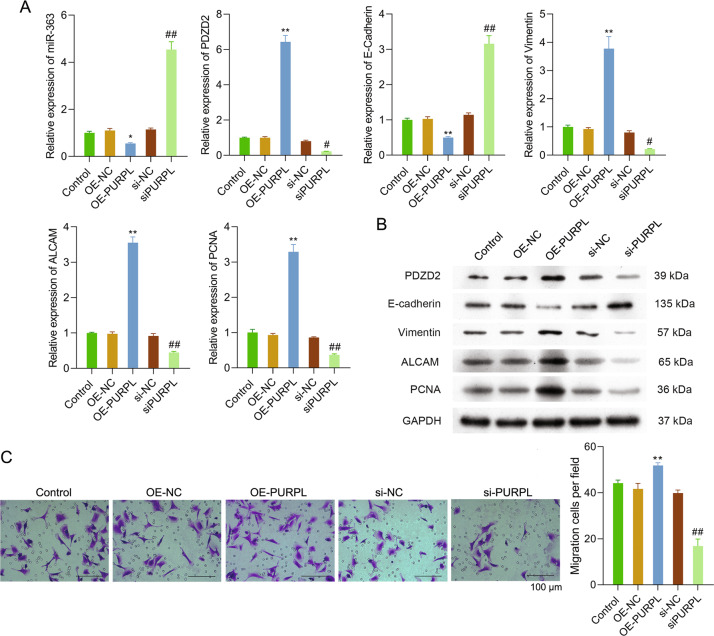
PURPL attended to the modulation of MG-63 cell EMT and TAMs migration. OE-PURPL or si-PURPL was transfected into MG-63 cells. **A** qPCR was applied to test miR-363, PDZD2, E-cadherin, vimentin, ALCAM, and PCNA expressions in MG-63 cells. **B** The protein expressions of PDZD2, E-cadherin, vimentin, ALCAM, and PCNA were measured using western blotting. **C** TAMs were cultivated into the above chamber, while MG-63 cells were put into the below chamber. Forty-eight hours later, the migration of TAMs was tested. *P < 0.05, **P < 0.01 vs. OE-NC group. ^*#*^*P* < 0.05, ^*##*^*P* < 0.01 vs. si-NC group.

To explore the influence of PURPL overexpression or knockdown on MG-63 cell EMT, as well as miR-363 and PDZD2 expression, further experiments were used. Figure 4A showed that the miR-363 and E-cadherin expressions (*P* < 0.01) were reduced by OE-PURPL transfection, but PDZD2, vimentin, ALCAM, and PCNA expressions in MG-63 cells were enhanced (*P* < 0.01). Opposite effects were seen due to si-PURPL transfection (*P* < 0.01). After OE-PURPL or si-PURPL transfection, the protein levels of PDZD2, E-cadherin, vimentin, ALCAM, and PCNA showed a similar tendency (Fig. 4B). As shown in these results, PURPL also attended to the modulation of MG-63 cell EMT, as well as miR-363 and PDZD2 expression.

### MG-63 cells with abnormal expression of PURPL influenced TAMs migration

After PURPL overexpression or knockdown in MG-63 cells, we also evaluated the TAMs migration. Figure 4C displayed that MG-63 cells with OE-PURPL transfection promoted the TAMs migration, as seen by the increased number of migrated TAMs in the OE-PURPL group (*P* < 0.01). si-PURPL transfection inhibited the TAMs migration showing contrary influence (*P* < 0.01). Thus, MG-63 cells with abnormal expression of PURPL influenced TAMs migration.

### Silence of PDZD2 weakened the influence of PURPL overexpression on MG-63 cell function

**Fig. 5:**
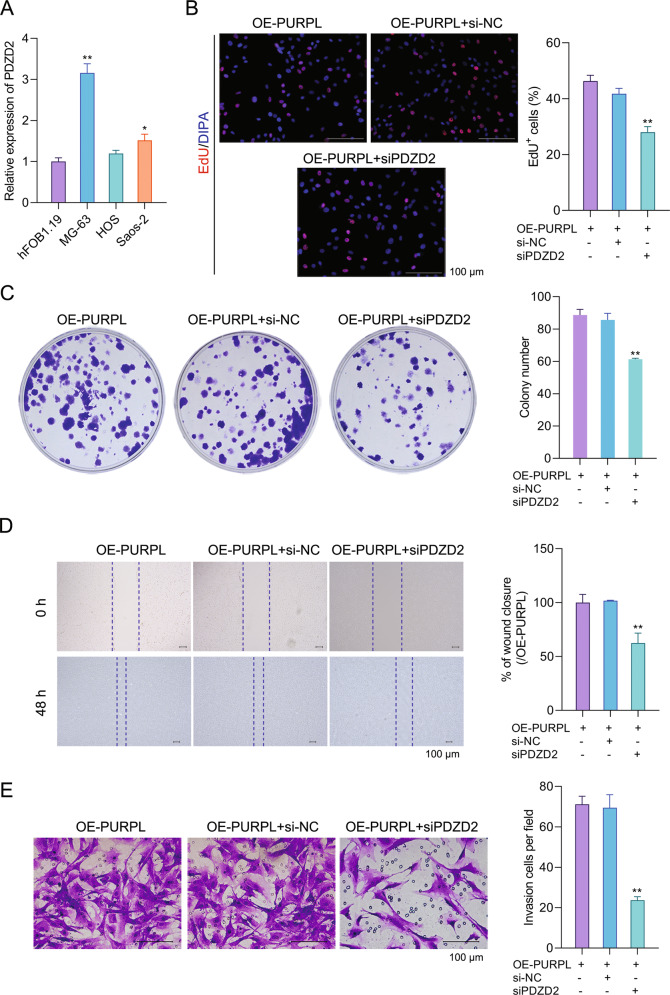
Silence of PDZD2 weakened the influence of PURPL overexpression on MG-63 cell proliferation, migration, and invasion. **A** qPCR was used to test the PDZD2 expression in hFOB1.19, MG-63, HOS, and Saos-2 cells. MG-63 cells were subjected to OE-PURPL and/or siPDZD2 transfection. Proliferation (**B**, **C**), migration (**D**), and invasion (**E**) of MG-63 cells were detected via EdU incorporation assay, colony formation assay, scratch wound assay, and transwell assay, respectively. *P < 0.05, **P < 0.01 vs. hFOB1.19 or OE-PURPL + si-NC group.

**Fig. 6:**
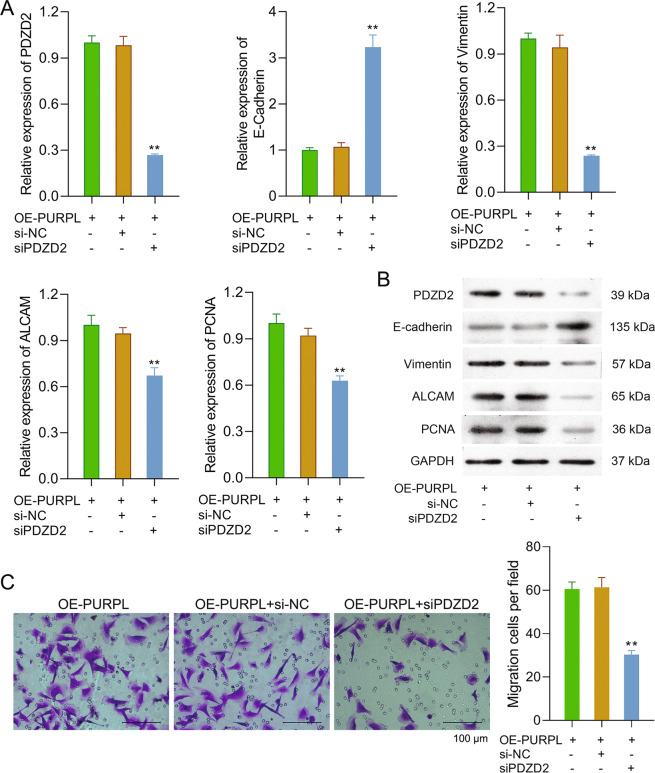
Silence of PDZD2 weakened the influence of PURPL overexpression on MG-63 cell EMT and TAMs migration. MG-63 cells were subjected to OE-PURPL and/or siPDZD2 transfection. **A**, **B** The mRNA and protein expressions of PDZD2, E-cadherin, vimentin, ALCAM, and PCNA in MG-63 cells were tested via qPCR and western blotting. **C** TAMs were cultivated into the upper- chamber, while MG-63 cells were put into the lower- chamber. Forty-eight hours later, the migration of TAMs was tested. **P < 0.01 *vs*. OE-PURPL + si-NC group.

As shown in Fig. 5A, compared to hFOB1.19 cells, PDZD2 expression was increased in MG-63 and Saos-2 cells (*P* < 0.05 or *P* < 0.01). To explore whether PURPL regulated MG-63 cell function via the miR-363/PDZD2 axis, siPDZD2 was transfected. As evidenced by the decreased number of EdU^+^ cells and colony number (*P* < 0.01), Fig. 5B, C were displayed to the OE-PURPL + si-NC group, cell proliferation was clearly inhibited in the OE-PURPL + siPDZD2 group. Figure 5D, E showed that relative to the OE-PURPL + si-NC group, the percentage of wound closure and invasion cells per field notably decreased in the OE-PURPL + siPDZD2 group (*P* < 0.01). This showed that siPDZD2 transfection also suppressed MG-63 cell migration and invasion. Moreover, as shown in Fig. 6A, B, the PDZD2, vimentin, ALCAM, and PCNA expressions in MG-63 cells were reduced, but the E-cadherin expression was raised in the OE-PURPL + siPDZD2 group compared to the OE-PURPL + si-NC group (*P* < 0.01 in mRNA level). As suggested by these results, the silence of PDZD2 weakened the influences of PURPL overexpression on MG-63 cell function.

### Silence of PDZD2 weakened the effects of PURPL overexpression on TAMs migration

We explored the influence of PDZD2 silence on PURPL overexpression-induced increase of TAMs migration. As shown in Fig. 6C, compared to the OE-PURPL + si-NC group, the migrated TAMs in the OE-PURPL + siPDZD2 group were remarkably low (*P* < 0.01), which suggested that the silence of PDZD2 weakened the effects of PURPL overexpression on TAMs migration.

## Discussion

Being a common and rapid-progressing bone tumor, OS affects the health and life of many patients [20]. The major grounds for the unsatisfied prognosis of patients with OS were tumor recurrence and metastasis [20, 21]. TAMs have been widely demonstrated to be implicated in the progression of OS, which can infiltrate into the OS tissue and promote OS immunosuppression, invasion, and metastasis [22]. To save lives, more understanding of the mechanisms of TAMs on OS progression will be useful for slowing the progression of OS [23, 24]. In recent years, a variety of non-coding RNAs have been discovered in cells [25]. They are proved to join in the regulation of multiple cell functions, including tumorigenesis [25, 26]. As reported in previous literature works, lncRNA UCA1 and lncRNA RP11-361F15.2 were associated with the tumor-promoting function of TAMs on OS progression [11, 12]. LncRNA PURPL contributed to the regulation of the p53 level in cells [13]. As proved in earlier studies, PURPL played a tumor-promoting role in colorectal cancer [13], gastric cancer [15], and liver cancer [27]. In this research, the PURPL expression in OS cells was upregulated by TAMs. PURPL overexpression accelerated OS cell proliferation, migration, invasion, and EMT, whereas PURPL knockdown had contrary influences. As indicated by these findings, TAMs facilitated OS growth, and metastasis might be achieved via enhancing PURPL expression. Besides, lncRNA MALAT1, lncRNA XIST, and lncRNA NORAD were reported to predict poor survival in OS patients [28–30]. We discovered that these three lncRNAs were also upregulated in OS cells after TAMs co-culture, which implied that there were multiplex lncRNAs involved in regulating TAMs on the OS development may jointly constitute a complex regulatory network.

The key downstream effectors of lncRNAs in cells are miRs [19]. They can combine with mRNAs and lead to gene expression silencing in cells [31]. As reported by Syed et al. [32], the immune escape mechanism of sarcoma cells was also related to miRs. To kidnap macrophages, tumor cells can release miRs and thereby lead them to exhibit a tumor-promoting role [32]. miR-363 was demonstrated to suppress multiple cancer progression as a well-studied tumor-suppressive miR [33, 34]. Our earlier experiment revealed that in OS cells, miR-363 was lowly expressed [17]. The overexpression of miR-363 repressed OS cell proliferation, migration, invasion, and EMT, as well as promoted cell apoptosis and G1/S arrest [17]. Herein, we discovered that PURPL bound to miR-363 and negative-modulated miR-363 expression in OS cells, which indicated that miR-363 was the downstream effector of PURPL. We also revealed that PURPL in OS cells positively regulated TAMs migration, while miR-363 in OS cells reversely regulated TAMs migration. As indicated by these findings, the PURPL/miR-363 axis promoted OS progression also via facilitating TAMs migration.

Polytropic protein interrelation modules that can recognize the internal and carboxy-terminal peptide motifs are PDZ domains [35]. PDZD2 is discovered to contain six PDZ domains [35]. PDZD2 was proved as a downstream effector of miR-363 in OS cells in an earlier literature [17]. The knockdown of PDZD2 restrained OS cell proliferation, migration, and invasion but enhanced cell apoptosis [17]. We found that PURPL positively regulated PDZD2 expression in OS cells in this research. Moreover, the effects of PURPL overexpression on OS cell proliferation, migration, invasion, and EMT, as well as TAMs migration, were weakened due to the silence of PDZD2. These findings indicated that the PURPL/miR-363 axis was targeted to PDZD2 to regulate OS growth and metastasis, as well as TAMs migration.

**Fig. 7:**
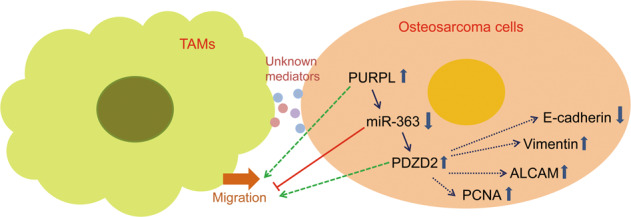
The interrelation of TAMs and PURPL/miR-363/PDZD2 axis on OS progression. TAMs-promoted OS progression could be through regulating PURPL/miR-363/PDZD2 signaling. PURPL/miR-363/PDZD2 aix also could feedback modulate TAMs migration.

Thus, this research affirmed that TAMs-promoted OS progression could be through regulating PURPL expression, thereby influencing miR-363/PDZD2 signaling. In addition, the PURPL/miR-363/PDZD2 axis also could modulate TAMs migration. According to our research outcomes, the putative interrelation of TAMs and the PURPL/miR-363/PDZD2 axis with OS progression was shown in Fig. 7. In the future, more research works should be carried out to investigate the feedback modulation and crosstalk between TAM and osteosarcoma cells.

## Materials and methods

### Cell culture and TAM-like macrophage induction

CD14^+^ peripheral blood mononuclear cells (PBMCs) were isolated from human peripheral blood using the auto-MACS Pro Separator (Miltenyi Biotec GmbH, Germany). Informed consents were obtained from five volunteers (healthy male, aged 20–30 years). The ethics approval (IRB# NO.202107004) was stamped by the Ethics Committee of Hangzhou First People’s Hospital, which was conducted in accordance with the Declaration of Helsinki (as revised in 2013). MG-63, HOS, and Saos-2 cells were purchased and authenticated by National collection of authenticated cell cultures, and CD14^+^ PBMCs were grown in high glucose MEM (Gibco, CA, USA), replenishing 10% fetal bovine serum (FBS, Gibco), 1% penicillin (Genview, FL, USA), and 1% streptomycin (Genview). Normal human osteoblastic hFOB1.19 cells were grown in DMEM/Ham’s F12 medium (DMEM/F12; 1:1 w/w mix) replenishing 10% FBS, 1% penicillin, 1% streptomycin, and 300 μg/mL neomycin.

### TAM-like macrophage induction

Similar to the literature reported earlier, an osteosarcoma-conditioned medium was prepared [36]. A total of 5 × 10^6^ MG-63 cells were cultivated in 100 mm dishes with 10 mL culture medium for 24 h. Using high glucose MEM containing 10% human AB serum (Gibco) for 48 h, the culture medium was changed. Then, the supernatants were collected, centrifuged, and stored.

To induce TAM-like macrophages, CD14^+^ PBMCs were stimulated by 25 ng/mL human macrophage colony-stimulating factor (hM-CSF, Sigma, MO, USA) for 6 days and then incubated with 50% osteosarcoma-conditioned medium for 2 days.

### Real-time PCR

In MG-63 cells, the mRNA expressions of CD68, CD163, CD204, IL-10, CCL1, PDZD2, E-cadherin, vimentin, activated leukocyte cell adhesion molecule (ALCAM). Proliferating cell nuclear antigen (PCNA), as well as the expressions of lncRNA metastasis-associated lung adenocarcinoma transcript 1 (MALAT1), lncRNA X-inactive specific transcript (XIST), lncRNA activated by DNA damage (NORAD), PURPL, and miR-363, were tested via real-time PCR. A Trizol solution was used to separate total RNAs (TAKARA, Japan). A Bestar^TM^ qPCR RT kit (DBI Bioscience, Shanghai, China) was used to composite cDNA. A Bestar^TM^ qPCR Master Mix (DBI Bioscience) was used to carry out RT qPCR. U6 and GAPDH played the roles of internal controls, and the 2^−△△Ct^ method was used to analyze the results. Primer’s information was displayed in Table 1.

**Table 1:** The primers information for real-time PCR.

Name	Sequence (5′-3′)
CD68 F	CTGTGCTTTTCTCGGGGG
CD68 R	CCGTGGCATTTCCATGACTA
CD163 F	CCACAAAAAGCCACAACAGG
CD163 R	GCAAGAATTCATCTCCCGGT
CD204 F	TGCTATCTCAGTGCAATCATCA
CD204 R	CGAGGAGGTAAAGGGCAATC
IL-10 F	CTGCCTAACATGCTTCGAGA
IL-10 R	CCTTGATGTCTGGGTCTTGG
CCL1 F	ATGGCATGGACTGTGGTCAT
CCL1 R	TGAACCCATCCAACTGTGTC
MALAT1 F	GCTCAAATCTTTCCACACGC
MALAT1 R	CACCGGAATTCGATCACCTT
XIST F	TTTCTTACTCTCTCGGGGCT
XIST R	TACGCCATAAAGGGTGTTGG
NORAD F	GAGAGACGCAGAACGCA
NORAD R	GGGCAGCCACAGCAG
PURPL F	TGTGAATTTAGGCCTACGTGA
PURPL R	AGCCCCTCATTCGTTATATTTT
miR-363 F	CTCAACTGGTGTCGTGGAGTCGCAATTCAGTTGAG AAATTGC
miR-363 R	ACACTCCAGCTGGGCGGGTGGATCACGATGCA
PDZD2 F	AATCAATCACGGTCCACAGG
PDZD2 R	CAGCCACACTTCTCCCAATG
E-cadherin F	ATTTTTCCCTCGACACCCGAT
E-cadherin R	TCCCAGGCGTAGACCAAGA
Vimentin F	GAGAACTTTGCCGTTGAAGC
Vimentin R	GGCAGAGAAATCCTGCTCTC
ALCAM F	ACTTGACGTACCTCAGAATCTCA
ALCAM R	CATCGTCGTACTGCACACTTT
PCNA F	TTTCCTGTGCAAAAGACGGA
PCNA R	CCGTTGAAGAGAGTGGAGTG
GAPDH F	TGTTCGTCATGGGTGTGAAC
GAPDH R	ATGGCATGGACTGTGGTCAT
U6 F	CTCGCTTCGGCAGCACA
U6 R	AACGCTTCACGAATTTGCGT

### Cell transfection

To form PURPL overexpression plasmid (OE-PURPL), the full length of the PURPL gene was cloned into the pcDNA3.1 vector (Genechem Corporation, Shanghai, China). Short interfering RNA (siRNA) against PURPL (si-PURPL, 5′-CCCUCUUGCUUUGCAAAUAUU-3′), siRNA against PDZD2 (siPDZD2, 5′-CUCUGAACCAGGAGAAACAUU-3′), miR-363 mimics (5′-AAUUGCACGGUAUCCAUCUGUA-3′), and miR-363 inhibitor (5′-CAGAUGGAUACCGUGCAAUUUU-3′) were all supplied by Genechem Corporation. For cell transfection, Lipofectamine^TM^ 2000 Reagent (Invitrogen, CA, USA) was applied. Empty pcDNA3.1 vector scrambled siRNA and miR sequence served as negative control (NC).

### TAMs migration assay

A two-chamber transwell assay was used to detect the migration of TAMs (Costor, CA, USA). In the upper-chamber, 2 × 10^4^ TAMs in 100 μL FBS-free MEM were cultivated. In the lower- chamber, 2 × 10^4^ transfected or non-transfected MG-63 cells in 700 μL MEM were supplemented. Cells in the above chamber were removed carefully 48 h later using a cotton swab. After washing with phosphate buffer saline (PBS) and fixing with 4% paraformaldehyde, a crystal violet solution was used to dye the cells. To remove the membrane from the chamber, a scalpel was used. The membrane attached to TAMs was dried and sealed with a neutral resin. An OLYMPUS CX41 upright microscope was used to obtain the images (Tokyo, Japan). Six random fields were collected, and IPP software was used to count the cell number.

### Dual-luciferase reporter assay

A dual-luciferase reporter assay was used to verify the potential binding connection of PURPL and miR-363. By inserting the PURPL sequence, including the possible or mutated miR-363 binding site into the psiCHECK-2 plasmid (Promega, WI, USA), the PURPL-wide type (WT) reporter vector and the PURPL-mutated type (MUT) reporter vector (GTGCAAT was mutated to CGATGTC) were constructed. Then, MG-63 cells were co-transfected with PURPL-WT (or PURPL-MUT) plasmids and miR-363 mimics. The relative luciferase activity was detected 48 h later by a Dual-Luciferase Reporter Assay System (Promega). Results were presented as Renilla luciferase signal/Firefly luciferase signal (R/F).

### Cell proliferation assay

EdU incorporation assay (RIBOBIO, Guangzhou, China) and colony formation assay were used to test cell proliferation. For the EdU incorporation assay, we cultivated 1 × 10^4^ MG-63 cells in a 96-well plate and were exposed to relevant transfection. After 48 h of culture, the culture medium was changed with 150 μL of EdU solution (50 μM). Cells were fixed with 4% paraformaldehyde 2 h later and dyed for 30 min using an Apollo solution protected from light. DAPI was used to dye the cell’s nucleus. An inverted fluorescence microscope (Leica, Germany) was used to obtain the images. We also calculated EdU positive (+) cells (%).

After different transfections, 200 MG-63 cells in 300 μL MEM were cultivated in a 35 mm petri dish for 2–3 weeks at 37 °C for colony formation assay. On finding visible clones in the petri dish, MEM was discarded, and PBS was used to wash cells. Cells were dyed with a crystal violet solution after fixing with 4% paraformaldehyde. We used a microscope to count the colony number.

### Cell scratch assay

A six-well plate was used to cultivate 5 × 10^5^ MG-63 cells, and then these were exposed to relevant transfection. A 200 μL pipette was used to remove cells, which generated scratches. After washing with PBS, cells were cultured in FBS-free MEM at 37 °C. Images of scratches were recorded 48 h later via an inverted microscope (MOTIC, Xiamen, China). IPP software was used to analyze results.

### Cell invasion assay

Similarly, MG-63 cell invasion was detected except for the following three differences: (1) the membrane was precoated with Matrigel (Corning, NY, USA); (2) MG-63 cells were cultivated in the upper chamber; (3) 700 μL MEM (no cells) was put into the lower chamber.

### Western blotting

We used a lysis buffer to isolate total proteins in MG-63 cells (Beyotime Biotechnology, Shanghai, China). We used the BSA assay to test the protein concentration (Amresco, OH, USA). Then, in equal concentration, SDS-PAGE was carried out with proteins as described earlier in literature 14. Abcam Biotechnology (MA, USA) supplied anti-PDZD2 antibody (ab133324, 1:1000), anti-ALCAM antibody (ab109215, 1:1000), and anti-GAPDH antibody (ab8245, 1:10000). Cell Signaling Technology (MA, USA) supplied anti-E-cadherin antibody (#3195, 1:1000), anti-vimentin antibody (#35741, 1:10000), and anti-PCNA antibody (#13110, 1:1000). The enhanced chemiluminescence technique was used to visualize the bands of proteins.

### Statistical analysis

We used GraphPad Prism software to conduct Statistical analysis. From three repeated experiments, data were represented as mean ± standard deviation (SD). A Student’s *t*-test or one-way analysis of variance (ANOVA) was utilized for calculating *P* values. *P* ˂ 0.05 was considered as a significant difference.

## Supplementary information

Author Contribution Form

## Data Availability

The datasets supporting the conclusions of this article are included within the article.
