# Supplementary material for: Effect of tumor-associated macrophages on lncRNA PURPL/miR-363/PDZD2 axis in osteosarcoma cells
Source: Cell Death Discov. 2021 Oct 22;7:307. doi: 10.1038/s41420-021-00700-z (PMC8536668; doi:10.1038/s41420-021-00700-z)
Supplement: Supplementary file 1 — Author Contribution Form [file 41420_2021_700_MOESM1_ESM.pdf]

DECLARATION OF CONTRIBUTIONS TO ARTICLE

ADMC

Manuscript Number:	Journal Name:	
	Cell Death Discovery	(the 'Journal')

Proposed Title of the Contribution:

Effect of tumor-associated macrophages on lncRNA PURPL/miR-363/PDZD2 axis in osteosarcoma cells (the 'Contribution')

Author(s):

Fan He, Guoming Ding, Wu Jiang, Xiaoliang Fan, Liulong Zhu (the 'Authors')

For all *CDDis*covery articles, each person named as an author in the published version must be able to show he or she has contributed substantially to the article.

Authorship credit should be based on 1) substantial contributions to conception and design, acquisition of data, or analysis and interpretation of data; 2) drafting the article or revising it critically for important intellectual content; and 3) final approval of the version to be published. Authors should meet conditions 1, 2 and 3.

Any person who cannot be shown to have made a substantial contribution to the article cannot be listed as an author in the final version. The name of any person who is deemed to have made a minor contribution can, however, appear in the Acknowledgments section of the article.

Please complete the table below to indicate the contributions of all named authors to the manuscript.

Author Full Name:	Specification of Contribution to the Manuscript:
Fan He	Conception and design, Financial support, Manuscript writing.
Guoming Ding	Conception and design, Financial support, Manuscript writing.
Wu Jiang	Administrative support, Collection of data and analysis, Visualization.
Xiaoliang Fan	Administrative support, Collection of data and analysis, Visualization.
Liulong Zhu	Administrative support, Collection of data and analysis, Visualization.

Please complete the table below to indicate the contributions of all named authors to the figures.

Figure 1:

Fan He, Guoming Ding, Wu Jiang, Xiaoliang Fan and Liulong Zhu contributied equally t o figure 1. All authors participated in the typesetting and review of figure 1.

Figure 2:

Fan He, Guoming Ding, Wu Jiang, Xiaoliang Fan and Liulong Zhu contributied equally t o figure 2. All authors participated in the typesetting and review of figure 2.

Figure 3:

Fan He, Guoming Ding, Wu Jiang, Xiaoliang Fan and Liulong Zhu contributied equally t o figure 3. All authors participated in the typesetting and review of figure 3.

Figure 4:

Fan He, Guoming Ding, Wu Jiang, Xiaoliang Fan and Liulong Zhu contributied equally to figure 4. All authors participated in the typesetting and review of figure 4.

Figure 5:

Fan He, Guoming Ding, Wu Jiang, Xiaoliang Fan and Liulong Zhu contributied equally to figure 5. All authors participated in the typesetting and review of figure 5.

Figure 6:

Fan He, Guoming Ding, Wu Jiang, Xiaoliang Fan and Liulong Zhu contributied equally t o figure 6. All authors participated in the typesetting and review of figure 6.

Signed for and on behalf of the Author(s):	Print Name:	Date:
Fan He 2021.8.13	Fan He	20210813
